# The Story of the Insane from Year to Year

**Published:** 1903-01-03

**Authors:** 


### THE STORY OF THE INSANE FROM YEAR TO YEAR.

### FIFTEENTH ANNUAL SERIES.

### ROXBURGH DISTRICT ASYLUM.

HERE there was only a small increase in the numbers during the year, there being 311 at the beginning and 317 at the end of it. There were 33 first admissions, 15 re-admissions, 20 discharged recovered, 11 discharged relieved, and 11 died. The average daily number resident was 313, and the total number under treatment was 356. The recoveries stand at 41·6 per cent. of the admissions, and the deaths at 3·5 per cent. of the daily number resident, the former being rather high, and the latter being very low. Of the year's deaths only three were caused by cerebral and spinal diseases, and only two by consumption. Of the year's admissions 15 were supposed to owe their insanity to moral causes, eight to intemperance in drink, 20 to hereditary influence, and 17 to previous attacks. The cause was ascertained in every instance except one. As is common in public asylums north of the Tweed, the Roxburgh one receives private patients, and the ordinary rate charged is £40 per annum, but when it is satisfactorily shown that such a sum cannot reasonably be afforded by the patient's friends a reduction in this rate is made. This is an enlightened policy to pursue, for it means a large number of patients can avoid the stigma of pauperism, and there is a proportionate diminution of the rate-supported cases. It is, however, much to be regretted that the number of beds available at Roxburgh is so limited. A few of our English asylums have provided annexes for private patients, but a few only, and until the provision is universal we cannot pride ourselves that our lunacy system is nearly perfect. There is in the report a comment on the vexed question of the increase or non-increase of insanity in our generation, and Dr. Johnstone says, "there is no reason to believe that this increase is due to a more active production of insanity in the district. The growth of the asylum population is simply due to the fact that the sum of each year's removals by death, recovery and otherwise, is less than the sum of the year's admissions." Dr. Johnstone's report is an unusually able and interesting one, and we feel sorry that space prevents our quoting it at greater extension; but we must find room for the following remarks on the causes of insanity. "In the great majority of cases the tendency of insanity is an inherited one; that is to say that in these cases the disease is purely the result of ill-assorted matrimonial unions. It would be idle to preach Draconian measures to a squeamish generation, which obstinately shuts its eyes to the plain teaching of nature; but it may be confidently asserted that, if parents and guardians would display only one-tenth part of that consideration for posterity which the common breeder of stock devotes to securing a sound and healthy strain, there would be no necessity to enlarge our asylums." This is indubitable, but why are these ill-assorted unions allowed to go on? The weekly cost per head is nearly 11s.

### MIDLAND COUNTIES IDIOT ASYLUM AT KNOWLE.

AT the beginning of the year there were 68 patients in residence, and 13 were admitted during the year, making a total of 81 under treatment. Of these 1 was discharged much improved, 1 moderately improved, 3 not improved, and 9 died. At the end of the year 67 remained under treatment. It is stated that "the increasing number of epileptic cases is becoming a source of anxiety. Although cases of confirmed epilepsy are not eligible, it seems hard to have them removed if the attacks should prove permanent." It is hoped that a separate wing may be erected for these cases in the near future, and we hope so too, but it is curious to notice how very slowly subscriptions come in for anything connected with an asylum. At this asylum there was a deficit during the year of £752, and only £404 was received in annual subscriptions to wipe off the deficit.

